# SSBD: an ecosystem for enhanced sharing and reuse of bioimaging data

**DOI:** 10.1093/nar/gkae860

**Published:** 2024-10-31

**Authors:** Koji Kyoda, Hiroya Itoga, Yuki Yamagata, Emi Fujisawa, Fangfang Wang, Miguel Miranda-Miranda, Haruna Yamamoto, Yasue Nakano, Yukako Tohsato, Shuichi Onami

**Affiliations:** Laboratory for Developmental Dynamics, RIKEN Center for Biosystems Dynamics Research, 2-2-3 Minatojima-minamimachi, Chuo-ku, Kobe, Hyogo 650-0047, Japan; Laboratory for Developmental Dynamics, RIKEN Center for Biosystems Dynamics Research, 2-2-3 Minatojima-minamimachi, Chuo-ku, Kobe, Hyogo 650-0047, Japan; Life Science Data Sharing Unit, RIKEN Information R&D and Strategy Headquarters, 2-2-3 Minatojima-minamimachi, Chuo-ku, Kobe, Hyogo 650-0047, Japan; Integrated Bioresource Information Division, RIKEN Bioresource Research Center, 3-1-1 Koyadai, Tsukuba, Ibaraki 350-0074, Japan; Laboratory for Developmental Dynamics, RIKEN Center for Biosystems Dynamics Research, 2-2-3 Minatojima-minamimachi, Chuo-ku, Kobe, Hyogo 650-0047, Japan; Laboratory for Developmental Dynamics, RIKEN Center for Biosystems Dynamics Research, 2-2-3 Minatojima-minamimachi, Chuo-ku, Kobe, Hyogo 650-0047, Japan; Life Science Data Sharing Unit, RIKEN Information R&D and Strategy Headquarters, 2-2-3 Minatojima-minamimachi, Chuo-ku, Kobe, Hyogo 650-0047, Japan; Laboratory for Developmental Dynamics, RIKEN Center for Biosystems Dynamics Research, 2-2-3 Minatojima-minamimachi, Chuo-ku, Kobe, Hyogo 650-0047, Japan; Laboratory for Developmental Dynamics, RIKEN Center for Biosystems Dynamics Research, 2-2-3 Minatojima-minamimachi, Chuo-ku, Kobe, Hyogo 650-0047, Japan; Laboratory for Developmental Dynamics, RIKEN Center for Biosystems Dynamics Research, 2-2-3 Minatojima-minamimachi, Chuo-ku, Kobe, Hyogo 650-0047, Japan; Laboratory for Developmental Dynamics, RIKEN Center for Biosystems Dynamics Research, 2-2-3 Minatojima-minamimachi, Chuo-ku, Kobe, Hyogo 650-0047, Japan; Faculty of Information Science and Engineering, Ritsumeikan University, 2-150 Iwakura-cho, Ibaraki, Osaka 567-8570, Japan; Laboratory for Developmental Dynamics, RIKEN Center for Biosystems Dynamics Research, 2-2-3 Minatojima-minamimachi, Chuo-ku, Kobe, Hyogo 650-0047, Japan; Life Science Data Sharing Unit, RIKEN Information R&D and Strategy Headquarters, 2-2-3 Minatojima-minamimachi, Chuo-ku, Kobe, Hyogo 650-0047, Japan

## Abstract

SSBD (https://ssbd.riken.jp) is a platform for the sharing and reuse of bioimaging data. As part of efforts to build a bioimaging data ecosystem, SSBD has recently been updated to a two-tiered data resource comprising SSBD:repository, a public repository for the sharing of all types of bioimaging data reported in journals, and SSBD:database, an added-value database for the sharing of curated, highly reusable, metadata-rich data. This update addresses the conflicting demands of rapid data publication and sharing of richly annotated data, thereby promoting bioimaging data sharing and reuse. With this update, SSBD is now positioned as a core repository and database within the foundingGIDE, an international consortium working to establish a global image data ecosystem. Harmonizing metadata between data resources enables cross-searching and data exchange with data resources from other countries and regions.

## Graphical Abstract

**Graphical Abstract:**
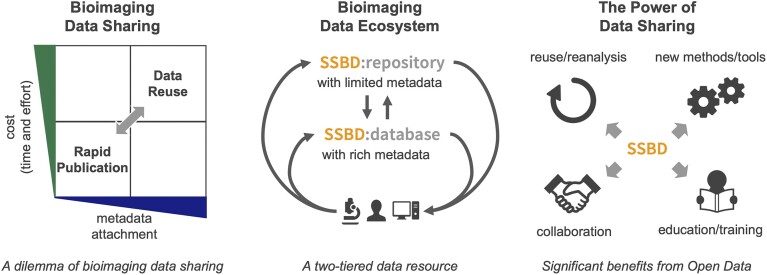

## Introduction

Bioimaging technologies allow us to capture biological phenomena as they are, both inside and outside cells. For this reason, alongside molecular biology technologies, these imaging technologies have become some of the most effective tools for understanding the mechanisms that underlie biological phenomena. In recent years, the importance of sharing bioimaging data according to the FAIR (Findable, Accessible, Interoperable and Reusable) principles (1), similar to nucleotide sequences, protein structures and gene expression data, has been recognized (2–4), leading to the establishment of several public data repositories and databases (5–8). In Europe, the BioImage Archive has been established as a public repository of imaging data, and the Image Data Resource and the Electron Microscopy Public Image Archive have been developed as added-value databases (AVDBs) of optical and electron microscopy data. In the United States, the establishment of similar public data resources has been extensively discussed (9).

SSBD is a platform for the sharing and reuse of bioimaging data that we develop with support from the National Bioscience Database Center of the Japan Science and Technology Agency (5). At its launch in 2013, SSBD was a public database designed primarily for the sharing of datasets containing original image data together with extracted biosystems dynamics data (e.g. segmentation and tracking data). However, in 2015, as part of efforts to advance image analysis technology and promote access to state-of-the-art imaging technologies through data sharing, the database was expanded to include stand-alone image data. Then, in 2016, in response to requests from authors, SSBD launched a repository service for the deposition of bioimaging data before article publication. These experiences laid the groundwork for the subsequent development of the bioimaging data ecosystem concept.

Recently, the international community has expressed the need for a bioimaging data ecosystem (2,3). This ecosystem would comprise two tiers: a repository for easy storage of datasets with limited metadata and an AVDB containing highly reusable, metadata-rich data. Such a two-tiered data resource would allow for rapid data publication, while also promoting the sharing and reuse of bioimaging data. In 2024, the foundingGIDE consortium was established to build a global image data ecosystem (GIDE) that facilitates the exchange of bioimaging data across the various data resources (https://founding-gide.eurobioimaging.eu).

## Bioimaging data ecosystem

**Figure 1.**
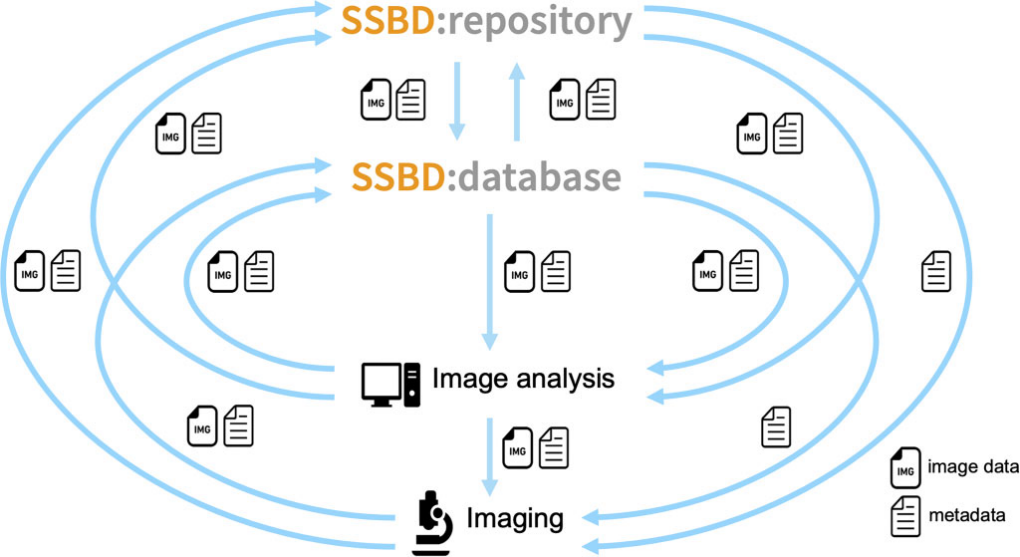
SSBD ecosystem for the sharing and reuse of bioimaging data. The system comprises two tiers: a repository tier (SSBD:repository) and an AVDB tier (SSBD:database). SSBD serves as a foundation for the sharing and reuse of bioimaging data, including image and analysis data, and metadata.

To build a bioimaging data ecosystem, we updated SSBD in 2019 to a two-tiered data resource comprising SSBD:repository and SSBD:database (Figure 1). SSBD:repository is a public repository that accepts and contains all types of bioimaging data reported in peer-reviewed articles. To enable rapid data publication, SSBD:repository allows authors to share bioimaging data by providing limited metadata such as contact information, licenses and species. Since the 2019 update, SSBD has also issued digital object identifiers that ensure persistent access to the deposited datasets.

**Figure 2.**
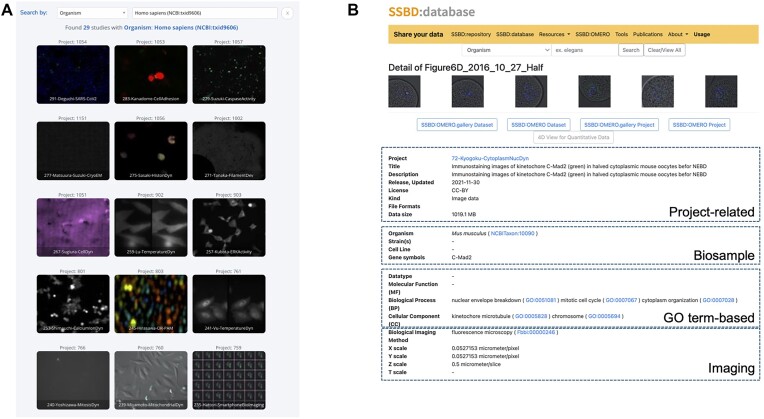
Representative screenshots of SSBD:database. (**A**) Example of bioimaging datasets deposited in SSBD:database. (**B**) Each dataset includes project-related metadata such as license information, biosample details, Gene Ontology (GO) annotations and imaging metadata.

SSBD:database is a public AVDB that contains highly reusable, metadata-rich data (Figure 2). In this context, ‘highly reusable’ refers to data that include images recorded by using state-of-the-art imaging technologies as well as data obtained through systematic experiments. Currently, expert curators select the data that are included in SSBD:database in two ways: by selecting image data that fit the above criteria from data already deposited in SSBD:repository or by identifying image data that fit the criteria from published papers. In both approaches, the curators assign metadata by using various ontologies (Table 1). After the curation process, the authors are asked to check the metadata before their data are published. In the future, the goal is to use SSBD:repository to gather all types of bioimaging data collected in, but not limited to, Japan and published in peer-reviewed journals; from these data, the curators will select those with the most extensive metadata to be shared in SSBD:database.

**Table 1.** Ontologies currently used or planned for future SSBD updates

Topic	Controlled vocabulary/ontology	RRID	Current	Planned
Biological processes, Cellular components, Molecular functions	Gene Ontology (GO) (26,27)	RRID:SCR_002811	✓	✓
Cell types	Cell Ontology (CL) (28)	RRID:SCR_004251		✓
Cell lines	Cell Line Ontology (CLO) (29)	RRID:SCR_005840	✓	✓
Anatomy	Uberon Multi-species Anatomy Ontology (UBERON) (30)	RRID:SCR_010668		✓
Imaging	Biological Imaging Methods Ontology (FBbi)	RRID:SCR_010235	✓	✓
Chemical compounds	Chemical Entities of Biological Interest Ontology (ChEBI) (31)	RRID:SCR_002088		✓
Organisms	NCBI Organismal Classification (NCBITaxon) (32)	RRID:SCR_000479	✓	✓
Experimental conditions	Ontology for Biomedical Investigations (OBI) (33)	RRID:SCR_006266		✓
Experimental conditions	Experimental Factor Ontology (EFO) (34)	RRID:SCR_003574		✓
Units	Units of Measurement Ontology (UO) (35)	RRID:SCR_010442		✓
Genes	Ensembl (ENSG) (36)	RRID:SCR_002344		✓
Proteins	Universal Protein Resource (UniProt) (37)	RRID:SCR_002380		✓
Controlled vocabulary	Medical Subject Headings (MeSH) (38)	RRID:SCR_004750	✓	✓

RRID: Research Resource Identifier (https://rrid.site/).

Recent advances in imaging technology have been rapid, with new techniques and equipment being developed in waves. As a result, the criteria for selecting highly reusable data are expected to evolve quickly. To address this issue, we are currently establishing an advisory board by inviting experts recommended by biological and biomedical societies in Japan. With insights from experts well-versed in bioimaging technologies across various fields, the SSBD:database will continuously update its inclusion criteria.

## Global sharing of bioimaging data

**Figure 3.**
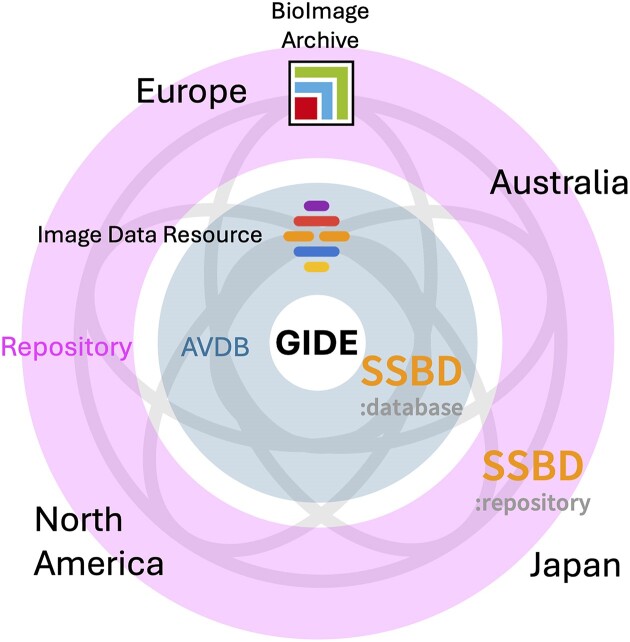
Overview of the proposed GIDE. Harmonizing the metadata used in each repository and AVDB enables the interoperability of shared bioimaging data across the various data resources.

The foundingGIDE consortium was established in 2024 to build a GIDE. Currently, the consortium consists of seven organizations from Europe, Japan and Australia. The plan is to build an ecosystem for the sharing of bioimaging and preclinical imaging data. SSBD is positioned within this ecosystem as a core repository and database. In addition, efforts are underway to harmonize the use of metadata among SSBD and the BioImage Archive and Image Data Resource in Europe. This will facilitate the exchange of bioimaging data across these different resources (Figure 3).

In the life sciences, global data sharing has already been achieved in several domains, such as the sharing of nucleotide sequences through the International Nucleotide Sequence Database Collaboration (10) and of protein structures through the Worldwide Protein Data Bank (11). Unlike the data shared in these fields, bioimaging data present a challenge because of their very large file sizes, making it impractical to mirror the data across multiple locations. Therefore, the global sharing for bioimaging data will be facilitated by harmonizing metadata, including information about the storage location of the bioimaging data. To achieve this harmonization, metadata must be standardized across the various bioimaging data resources through the use of controlled vocabularies and ontologies.

## Adoption of community guidelines and recommendations

In recent years, there has been an accelerated movement toward the standardization of metadata, with initiatives such as REMBI (Recommended Metadata for Biological Images) (12), which provides guidelines for metadata related to bioimaging data, and QUAREP-LiMi (the Consortium for Quality Assessment and Reproducibility for Instruments and Images in Light Microscopy) (13), which aims to improve the quality control and assurance of optical microscopy data. SSBD is undergoing major updates to align with the guidelines that have been published by these initiatives by adjusting the metadata structure and the components it uses and by using controlled vocabularies through various ontologies (https://doi.org/10.6084/m9.figshare.27023533). In addition to basic metadata such as contact information and licensing, we have been expanding the metadata to include items directly related to the data, such as species, biological processes, cellular components and molecular functions, by using various ontologies (Table 1). We have also added metadata related to the samples from which the data were collected, such as strain and cell line names, and metadata related to the imaging methods that were used. The overall intent of this metadata model update with controlled vocabularies is to ensure the consistency and accuracy of metadata.

Next-generation file format for storing bioimaging data has been proposed (14). In SSBD, we plan to provide all bioimaging data in OME-Zarr format (15). We have already shared 12 datasets (https://ssbd.riken.jp/ssbd-ome-ngff-samples) on S3-compatible object storage, enabling parallel data access; these datasets are intended to be used to develop and validate data visualization and analysis tools compatible with the OME-Zarr format and are expected to contribute to the widespread use of this format.

## Dataset update and reuse

**Figure 4.**
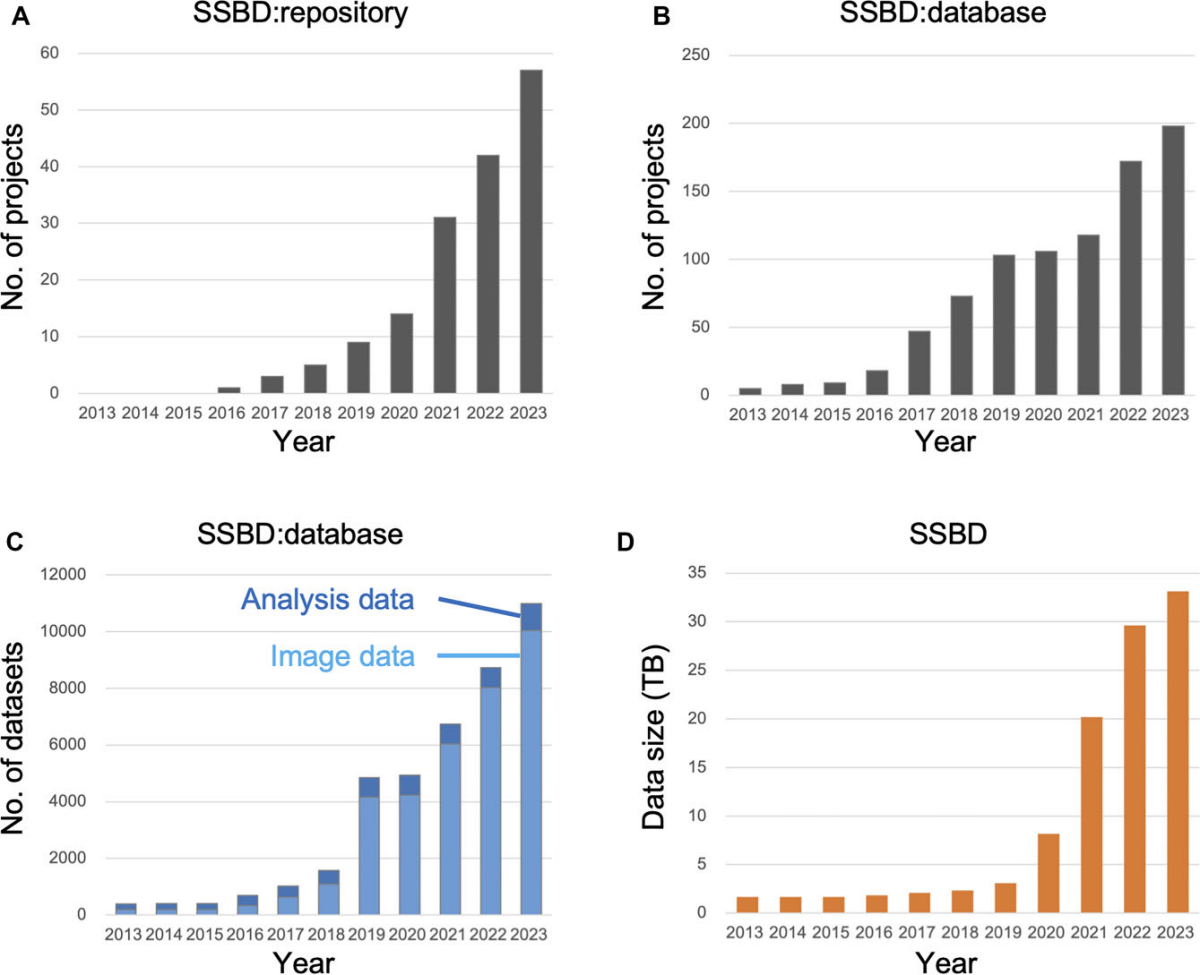
SSBD statistics. (**A**) Annual trend in the number of projects registered in SSBD:repository. (**B**) Annual trend in the number of projects registered in SSBD:database. (**C**) Annual trend in the number of datasets shared in SSBD:database. ‘Analysis data’ include segmentation and tracking data. (**D**) Total size of the data stored in SSBD.

The amounts of bioimaging data contained in SSBD have increased rapidly (Figure 4). Examples of datasets recently contained in SSBD include image data exceeding 100 million pixels captured with the trans-scale scope AMATERAS (16) and whole-brain imaging data of *Mus musculus* captured by using tissue clearing with light-sheet microscopy (17). Such datasets, captured by using state-of-the-art imaging technologies and covering a wide range of biological phenomena and tissues, are expected to lead to the development of new image analysis techniques and the discovery of new knowledge through data reuse. Similarly, images and morphological data concerning about 6000 gene mutants of the budding yeast *Saccharomyces cerevisiae* and phenotypic analysis results obtained through statistical analysis have also been contained in SSBD (18,19). These data, which comprise comprehensive image and morphological data for each of the genetic mutations, will be useful as reference data for future studies. Additionally, the comprehensive functional analysis of genes could be achieved through the development of new image analysis techniques.

A primary goal of SSBD is to facilitate the reuse of the data contained in its database. For example, SSBD can be used to build databases tailored to specific species, organs or phenomena. Two such databases have been built by using bioimaging data contained in SSBD. One is NeuroGT (20), a mouse-brain image database that enables the classification and manipulation of neuron subsets on the basis of neuronal birthdate. The other is AZEBEX (21), a database of brain gene expression in adult zebrafish. Therefore, by using SSBD, it is possible to build databases that combine metadata tailored to specific species, organs and phenomena, and to integrate bioimaging data with other data types.

We are also tracking the development of analytical techniques that have been developed by using data contained in SSBD. For example, a deep-learning-based method to extract anatomical regions from mouse brain image data has been developed, and the analysis data have been shared on SSBD (22). Also, a method has been created to detect phenotypic abnormalities by using variational auto-encoders from image segmentation data for nuclear regions in *Caenorhabditis elegans* embryos (23). Such analytical techniques developed by using data contained in SSBD will likely provide further data that will also be shared on SSBD.

## Dataset features

One of the features of SSBD is that it contains large amounts of biosystems dynamics data measured through image analysis. This is because SSBD has historically prioritized the collection of this type of data. Recently, numerous image segmentation methods have been developed with the rapid development of artificial intelligence technology, and the biosystems dynamics data contained in SSBD can be used to develop or evaluate the performance of these new image segmentation methods. SSBD shares its biosystems dynamics data in an open unified format, BD5 (24), that can be visualized by using QtBD5Viewer (https://doi.org/10.6084/m9.figshare.27023530). Another feature of SSBD is that it contains large amounts of time-series image data. This is because SSBD was originally developed for research focusing on the dynamics of biological phenomena. These data can contribute to the development of tracking methods.

We are also developing a new viewer to visualize image data synchronized with image segmentation and tracking data stored in SSBD. The new viewer will load image data stored in the OME-Zarr format by using the Viv library, along with biosystems dynamics data in BD5 format via the HDF5 Web API for visualization (Movie 1). We are also developing the BD-Zarr format, which will use OME-Zarr’s table extension to describe tracking data and the features of segmented biological objects (https://doi.org/10.6084/m9.figshare.27023527). In the near future, we plan to achieve more unified data access to further enable the visualization and analysis of image and biosystems dynamics data.

**Movie 1.**
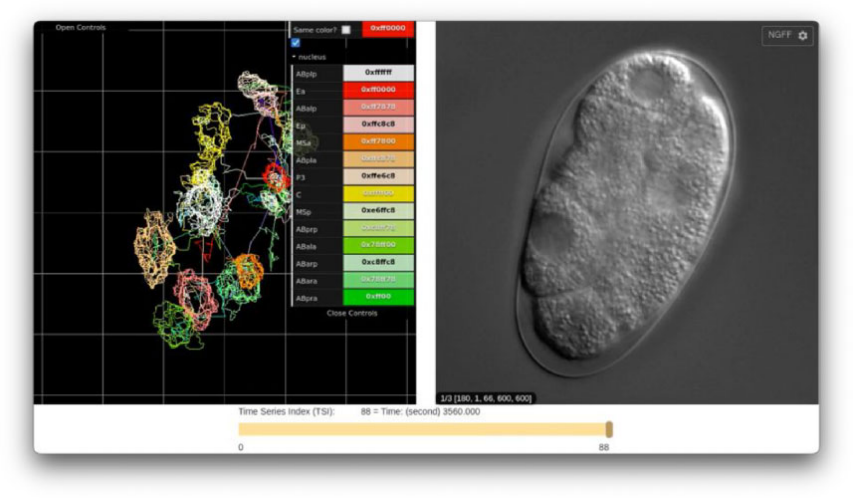
SSBD viewer (currently under development) will be able to synchronize and visualize image and biosystems dynamics data.

## Training and social media

To expand the number of users, we have been working to introduce researchers to the methods of depositing and using data in SSBD through seminars, webinars and training programs organized in collaboration with several domestic academic societies. Our ultimate goal, however, is to expand the use of SSBD by collaborating with bioimaging communities in other regions in the Asia–Pacific. Through foundingGIDE, collaborations with Australia’s preclinical imaging data resources have already begun. Through the holding of training programs and other initiatives, bioimaging data should be appropriately shared across the relevant data resources.

As part of our public-relations efforts, we actively use social media networks such as X and Instagram. On X (https://x.com/ssbd_en), we help maximize the impact of research from which data have been deposited in SSBD by posting links to data and introductions to the papers at publication. In addition, we are starting outreach activities through our Instagram account (https://www.instagram.com/bioimagram) to raise awareness of the importance of bioimaging technology research activities among a broader audience, including the general public. SSBD supports mobile phone and tablet access to facilitate direct access via these social media networks platforms.

## Outlook and challenges

We are currently planning to expand the range of data contained within SSBD. Recently, the development of techniques to measure spatial omics data has become increasingly active, with some of these methods being based on imaging technologies. SSBD is an ideal platform for sharing spatial omics data measured by these imaging techniques. The OME-Zarr community is also discussing how to store spatial omics data and share them in OME-Zarr format.

The aim of SSBD is not only to facilitate data sharing but also to promote data reuse. To achieve this, it will be necessary to share image analysis workflows and provide execution environments for the associated workflows. To support workflow sharing, we are currently collecting not only bioimaging data but also information about the storage locations of workflows on platforms such as GitHub from published papers. By establishing and introducing execution environments for these workflows, including external environments such as Google Colab and MyBinder, researchers can reproduce analyses and apply these workflows to their bioimaging data. These activities will allow SSBD to become a platform that covers the sharing and reuse of image and biosystems dynamics data and the reuse of workflows.

We are also working on the development of an image search service that uses image queries. By using methods such as WndChrm (25) and deep learning, we plan to classify the image data stored in SSBD and display images similar to the query image in ranked order. If the ranked image data contain links to workflows, it is expected that these workflows can also be applied to the query image data. In this way, SSBD can promote the reuse of workflows.

Finally, several tasks are underway to ensure sustainable database management. One of these tasks is web form-based data registration in SSBD:repository. Currently, data registration involves many manual steps to go from receiving an author’s request to data deposition. In the near future, with the implementation of government policies and prerequisites for data deposition before journal publication, data registration in SSBD will likely dramatically increase. By delegating the task of data registration to authors through web forms, we aim to reduce human error and improve efficiency. Another task that is underway is the automated annotation of metadata by using large language models. Currently, annotation in SSBD is performed manually, thus limiting the number of papers that can be handled each year (Figure 4B). Automated metadata annotation can assign predictive metadata to the data and assist curators in metadata assignment. By leveraging technologies such as large language models, we aim to reduce human- and time-related costs and thus establish a sustainable database management approach that can cope with the rapid growth of open data.

## Supplementary Material

gkae860_Supplemental_File

## Data Availability

All data and source codes are available on the SSBD (https://ssbd.riken.jp) and Figshare (https://doi.org/10.6084/m9.figshare.27023533, https://doi.org/10.6084/m9.figshare.27023530 and https://doi.org/10.6084/m9.figshare.27023527).
